# Mapping the structural, electrical, and optical properties of hydrothermally grown phosphorus-doped ZnO nanorods for optoelectronic device applications

**DOI:** 10.1186/s11671-019-2920-3

**Published:** 2019-03-28

**Authors:** Vantari Siva, Kwangwook Park, Min Seok Kim, Yeong Jae Kim, Gil Ju Lee, Min Jung Kim, Young Min Song

**Affiliations:** 1 School of Electrical Engineering and Computer Science, Gwangju Institute of Science and Technology, Gwangju, 61005 Republic of Korea; 2 Korea Advanced Nano Fab Center, Suwon, Gyeonggi-do 16229 Republic of Korea

**Keywords:** Phosphorus doping, P-type ZnO nanorods, Hydrothermal growth

## Abstract

The phosphorus-doped ZnO nanorods were prepared using hydrothermal process, whose structural modifications as a function of doping concentration were investigated using X-ray diffraction. The dopant concentration-dependent enhancement in length and diameter of the nanorods had established the phosphorus doping in ZnO nanorods. The gradual transformation in the type of conductivity as observed from the variation of carrier concentration and Hall coefficient had further confirmed the phosphorus doping. The modification of carrier concentration in the ZnO nanorods due to phosphorus doping was understood on the basis of the amphoteric nature of the phosphorus. The ZnO nanorods in the absence of phosphorus showed the photoluminescence (PL) in the range of the ultraviolet (UV) and visible regimes. The UV emission, i.e. near-band-edge emission of ZnO, was found to be red-shifted after the doping of phosphorus, which was attributed to donor-acceptor pair formation. The observed emissions in the visible regime were due to the deep level emissions that were aroused from various defects in ZnO. The Al-doped ZnO seed layer was found to be responsible for the observed near-infrared (NIR) emission. The PL emission in UV and visible regimes can cover a wide range of applications from biological to optoelectronic devices.

## Introduction

ZnO is one of the most promising semiconducting materials, which has received significant attention owing to its unique and easily tunable physical and chemical properties [1–11]. It is known that the ZnO is an intrinsic n-type semiconductor. The p-type conductivity in ZnO plays a key role in the formation of homojunction, which has several applications including light-emitting diodes [12], electrically pumped random lasers [2], and photodetectors [9]. Till now, several attempts were made to induce the p-type conductivity in the ZnO matrix by doping different elements such as antimony (Sb), arsenic (As), nitrogen (N), phosphorus (P), or other elements [2, 5–9]. However, few of these elements are likely to fail in inducing the p-type conductivity as they form deep acceptors and hence become not useful. The apparent bottleneck issues with the p-type doping in ZnO are the initial achievement and their reproducibility and stability [7]. Fortunately, the stability/degradation issues can be avoided in the case of phosphorus in ZnO by the thermal activation using rapid thermal annealing process [15]. Furthermore, the phosphorus-doped ZnO thin films were found to be stable up to 16 months under ambient conditions according to Allenic et al. [14]. Therefore, the phosphorus was considered to be one of the most reliable and stable ones for inducing the p-type conductivity in ZnO among the aforementioned dopants. Moreover, the phosphorus in ZnO nanostructures was found to trigger the oxygen vacancy-related photoluminescence (PL) emission in the visible region [8, 16]. Though there have been several reports on the PL emission study of ZnO nanostructures [17–22], a systematic study that can cover the luminescence in the three different and important regimes of the electromagnetic spectra including ultraviolet (UV), visible, and near-infrared (NIR) regimes along with their electrical and structural properties is quite scarce.

In the present study, we report the successful doping of phosphorus in ZnO nanorods using hydrothermal method, which is one of the cost-effective, scalable, large-area, and low-temperature techniques. The phosphorus was found to be amphoteric in nature, which was realized from an unconventional variation of the type of conductivity and carrier concentration as a function of the doping concentration. We further demonstrate the PL emission in the UV, visible, and NIR regions by controlled doping of phosphorus in the ZnO nanorods grown on Al-doped ZnO seed layer. The underlying mechanism of the present findings is discussed on the basis of various defect states in the existing system. The most interesting aspect of the present study is the achievement of emission in two different regimes (UV and visible) in a single system by carefully choosing an appropriate combination of the nanostructures, seed layer, and dopants.

## Methods

### Preparation of Seed Layer

**Fig. 1:**
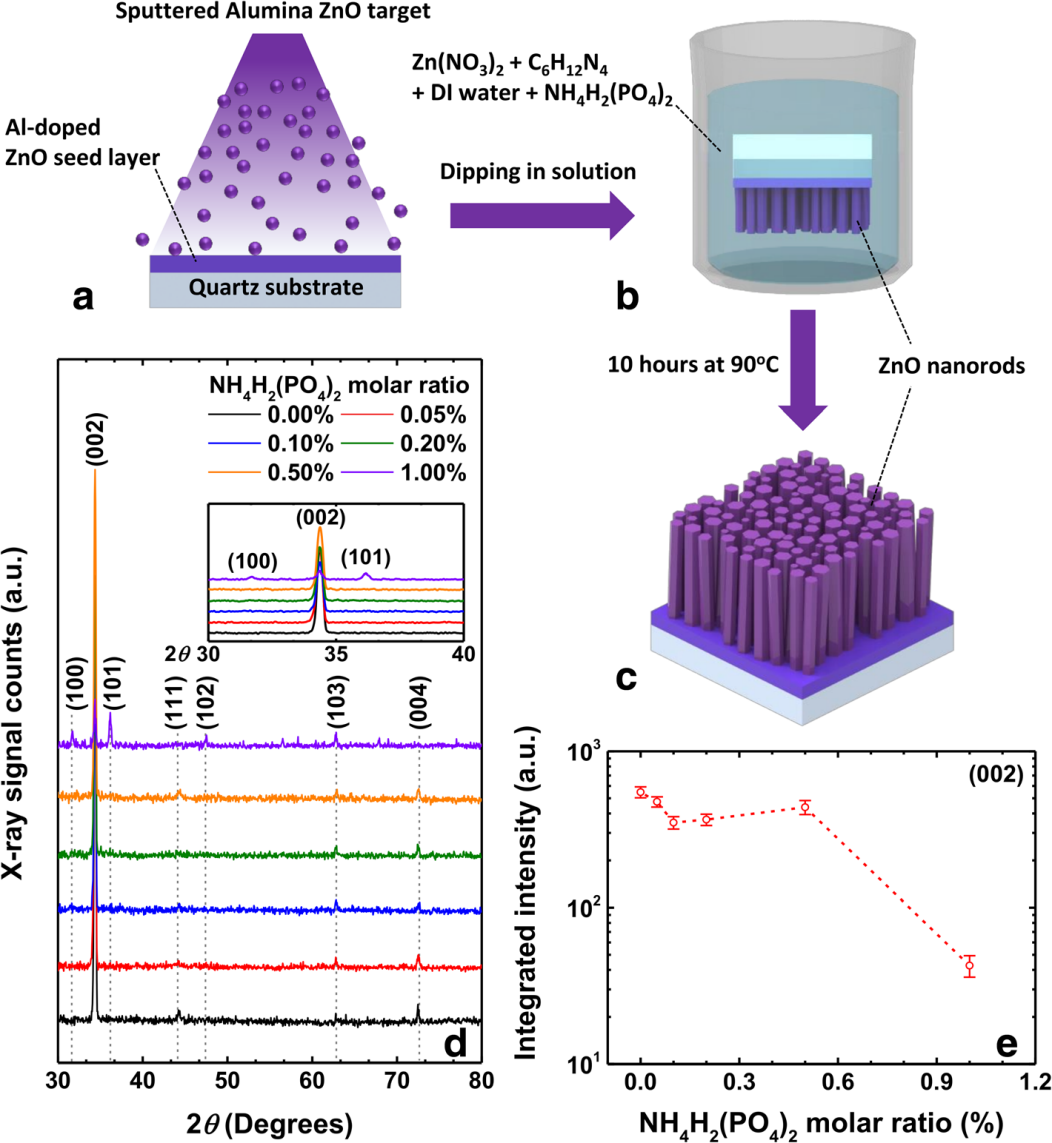
Schematic representation of the Al-doped ZnO seed layer (**a**), growth process of ZnO nanorods (**b**), and grown ZnO nanorods (**c**). The XRD patterns (**d**) of the ZnO nanorods corresponding to varying NH_4_H_2_(PO_4_)_2_ M ratio. The integrated intensity of (002) peak as functions of NH_4_H_2_(PO_4_)_2_ M ratio (**e**)

A seed layer of Al-doped ZnO film of approximately 100 nm was grown using radio frequency (RF) sputter deposition having a 2% of alumina ZnO target on a set of cleaned quartz substrates (Fig. 1a). The substrates were cleaned in acetone and isopropyl alcohol using ultrasonication, after which the substrates were dried carefully using nitrogen gas. The sputtering of the seed layer was carried out for 40 min using RF power of 90 W and 60 SCCM of Ar gas flow. The reason for choosing the Al-doped ZnO film as a seed layer was due to its better conductivity and more transmittance compared to pure ZnO film [23].

### Growth of ZnO Nanorods

The undoped ZnO nanorods were grown by hydrothermal method using zinc nitrate hexahydrate (Zn(NO_3_)_2_, reagent grade (98%), and hexamethylenetetramine (HMTA, C_6_H_12_N_4_, ≥ 99.0%). A solution of zinc nitrate and HMTA of 0.06 M in 400 ml volume were prepared by stirring for 2 h. The phosphorus-doped ZnO nanorods were prepared by adding ammonium dihydrogen phosphate (NH_4_H_2_(PO_4_)_2_, ≥ 98%) to the above chemicals in the M ratios of 0%, 0.05%, 0.1%, 0.2%, 0.5%, and 1%. The seed layer-deposited quartz substrates were dipped in these beakers and kept in the oven at 90 °C for 10 h (Fig. 1b). In next, these samples were rinsed with deionized water and thoroughly dried with nitrogen gas to arrive at the vertically aligned phosphorus-doped ZnO nanorods by removing the residues (Fig. 1c).

### Characterization Methods

The surface morphology of the samples was examined using a scanning electron microscope (SEM). The effect of doping on the structural properties of the samples was investigated using powder mode X-ray diffraction (XRD). Hall-effect measurements were performed on all the samples to understand the type of conductivity of the samples, where the magnetic field of 0.5 T has been applied. The room temperature PL measurements were performed using an excitation wavelength of 266 nm (Nd-YAG-pulsed laser) and incident power of 150 mW.

## Results and Discussion

In order to understand the structural changes due to the incorporation of phosphorus into ZnO nanorods, we performed the powder mode XRD measurements, whose plots are presented in Fig. 1d. We note here that the undoped sample shows the diffraction peaks at 34.36°, 44.27°, 62.80°, and 72.45° corresponding to (002), (111), (103), and (004) planes of ZnO, respectively. The peak corresponding to (002) plane shows the highest intensity, and the peak position does not change regardless of NH_4_H_2_(PO_4_)_2_ M ratio and its resulting diameter/length changes of the nanorods. Upon increasing the NH_4_H_2_(PO_4_)_2_ M ratio, the integrated intensity of the highest intensity peak, i.e., (002) peak, gradually decreases as shown in Fig. 1e. The only difference in these samples is the variation of the M ratio; hence, it can be attributed to the reduced crystalline nature of the ZnO nanorods [24]. However, one thing to note here is the full width at half maximum (FWHM) of the (002) peak. The FWHM was found to be nearly the same around 0.25° irrespective of the NH_4_H_2_(PO_4_)_2_ M ratio. In these perspectives, it is also highly likely that the misalignment of the nanorods in *c*-axis can also lead to the decrease in integrated (002) peak intensity. When the M ratio of NH_4_H_2_(PO_4_)_2_ reached to 1%, three additional peaks were observed at angles 31.70°, 36.17°, and 47.50°, which are related to (100), (101), and (102) peaks of ZnO crystal, respectively. The appearance of these additional peaks is also in good agreement with the abovementioned claim.

**Fig. 2:**
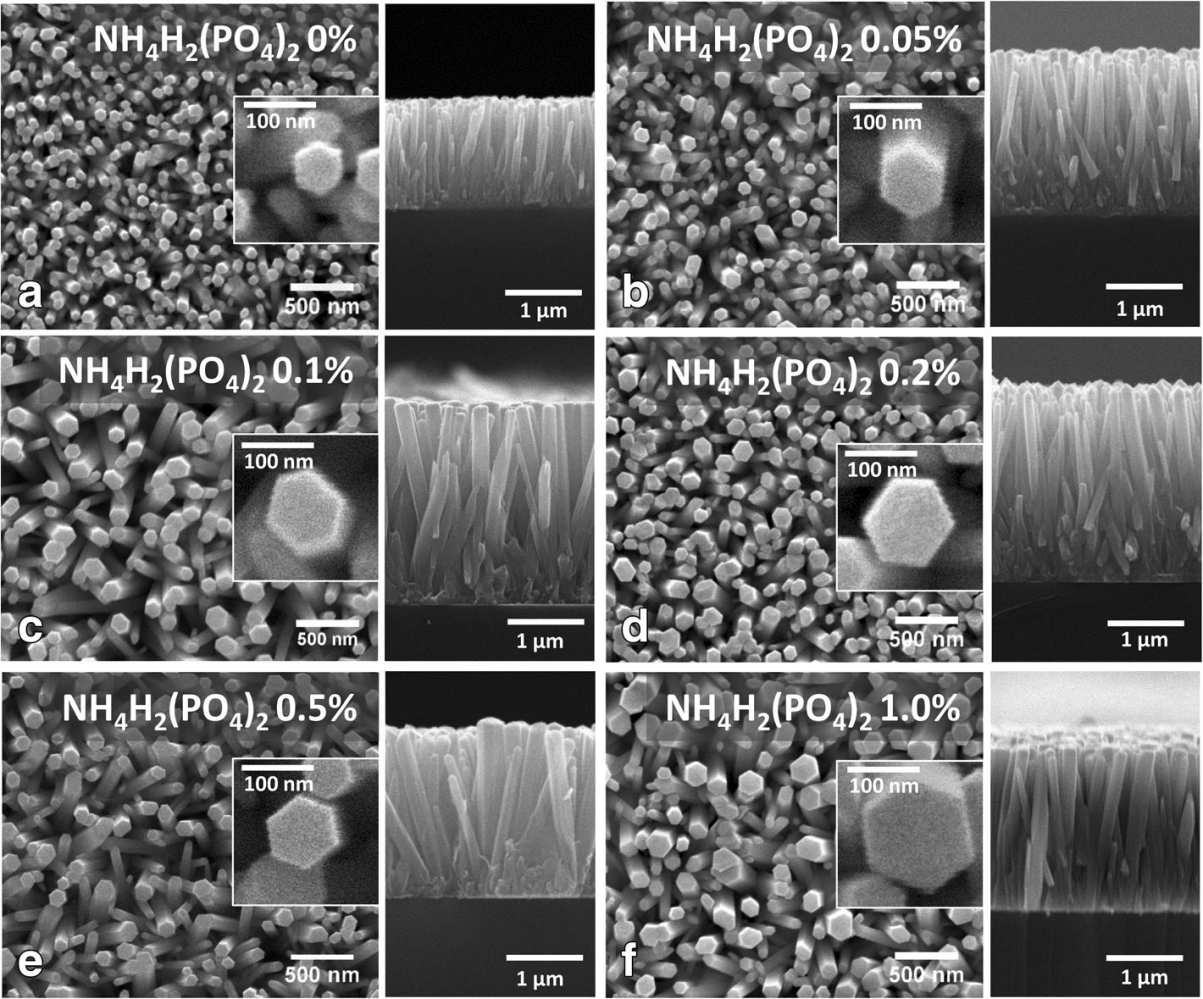
Top (left) and cross-sectional (right) SEM images of ZnO nanorods corresponding to NH_4_H_2_(PO_4_)_2_ M ratios 0% (**a**), 0.05% (**b**), 0.1% (**c**), 0.2% (**d**), 0.5% (**e**), and 1.0% (**f**), respectively. Diameter and length of the nanorods increased as functions of NH_4_H_2_(PO_4_)_2_ M ratio. The enhancement of volumetric features of the nanorods is due to the elevated incorporation of phosphorus

**Fig. 3:**
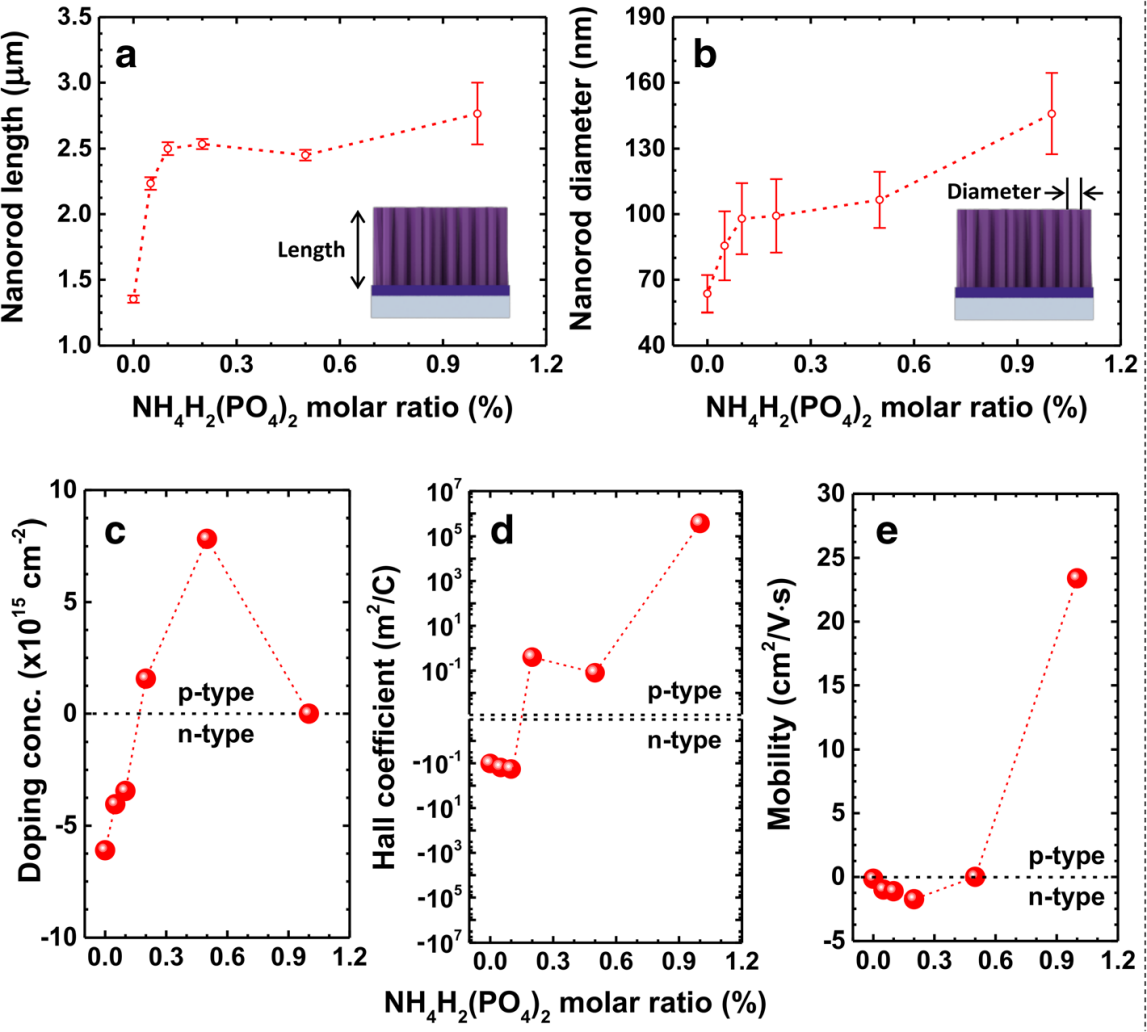
**a**, **b** Quantitative views of the length and diameter of ZnO nanorods with an increase of NH_4_H_2_(PO_4_)_2_ M ratio, respectively. **c**–**e** The changes in doping concentration, Hall coefficient, and mobility of the nanorods as functions of NH_4_H_2_(PO_4_)_2_ M ratio, respectively. Conductivity changed from negative to positive when NH_4_H_2_(PO_4_)_2_ M ratio is higher than 0.3% approximately. The decrease in the doping concentration of the nanorods corresponding to 1% of NH_4_H_2_(PO_4_)_2_ M ratio is due to the self-compensation effect beyond the solubility limit of phosphorus in the ZnO nanorods

The top and cross-sectional view of SEM images of the undoped and doped (up to 1%) samples are shown in Fig. 2a–f, where a uniform distribution of the hexagonal nanorods can be noticed. As discussed in the above paragraph, the diameter and length of the nanorods were found to be increased upon increasing the NH_4_H_2_(PO_4_)_2_ M ratio, which can be observed in the inset (top view) and right-hand side (cross-sectional view) of each image respectively. In the undoped sample (0% of NH_4_H_2_(PO_4_)_2_ M ratio), the average diameter of the nanorods was noticed to be approximately 60 nm, which kept on increasing gradually till 145 nm upon increasing the doping concentration as shown in the insets of Fig. 2a–f. Similarly, the length of the nanorods was also found to be increased with doping concentration though rather little increase as shown in the right-hand side of each image. The length and diameter of the nanorods are plotted as functions of NH_4_H_2_(PO_4_)_2_ M ratio in Fig. 3a and b, respectively. In the insets of these figures, we show the schematic illustration of vertically grown ZnO nanorods to indicate their length and diameters. It may be noted that the length of these nanorods also increases rapidly from 1.35 μm to 2.5 μm upon increasing the NH_4_H_2_(PO_4_)_2_ M ratio from 0% to 0.1% and almost saturates beyond this M ratio. A similar trend in the variation of diameter of the nanorods was noticed (Fig. 3b). The enhanced length and diameter of the nanorods till 0.1% of NH_4_H_2_(PO_4_)_2_ M ratio is attributed to the larger size of the phosphorus compared to oxygen atoms in the ZnO [12, 13, 25]. Beyond 0.1% M ratio, the nature of length and diameter variation can be understood on the basis of saturation of solubility limit of the incorporating phosphorus into ZnO matrix [26]. Though all the other parameters kept constant or slowed down to increase or decrease except the doping concentration, the length and diameter of the nanorods were still found to be increased, which indicates the successful incorporation of phosphorus into the ZnO nanorods [12, 25]. The chemical reactions responsible for the growth of ZnO and the doping of phosphorus into the ZnO crystals can be understood from the following equations [16]:

1 $$ \mathrm{Zn}{\left({\mathrm{NO}}_3\right)}_2\to {\mathrm{Zn}}^{2+}+2{\mathrm{NO}}_3^{-} $$

2 $$ {\mathrm{C}}_6{\mathrm{H}}_{12}{\mathrm{N}}_4+10{\mathrm{H}}_2\mathrm{O}\leftrightarrow 6\mathrm{HCHO}+4{{\mathrm{N}\mathrm{H}}_4}^{+}+4{\mathrm{OH}}^{-} $$

3 $$ {\mathrm{Zn}}^{2+}+2{\mathrm{OH}}^{-}\leftrightarrow \mathrm{Zn}{\left(\mathrm{OH}\right)}_2\to \mathrm{Zn}\mathrm{O}+{\mathrm{H}}_2\mathrm{O} $$

4 $$ \mathrm{N}{\mathrm{H}}_4{\mathrm{H}}_2\mathrm{P}{\mathrm{O}}_4+2{\mathrm{O}\mathrm{H}}^{-}\to {{\mathrm{NH}}_4}^{+}+2{\mathrm{H}}_2\mathrm{O}+{\mathrm{PO}}_4^{3-} $$

5 $$ 3{\mathrm{Zn}}^{2+}+2{{\mathrm{PO}}_4}^{3-}\to {\mathrm{Zn}}_3{\left({\mathrm{PO}}_4\right)}_2\downarrow $$

In the hydrothermal process, upon increasing the temperature, the zinc nitrate would decompose initially into Zn^2+^ and nitrate ions. On the other hand, the chemical reaction between HMTA and water molecule gives rise to formaldehyde, ammonium ions, and hydroxyl ions as shown above in Eq. (2). These hydroxyl ions react with the Zn^2+^ ions and lead to the formation of ZnO and H_2_O molecules. In addition, the ammonium dihydrogen phosphate reacts with the already existing hydroxyl ions in the beaker and forms phosphate ions along with ammonium ions and a water molecule. We note here that these phosphate ions react with the zinc ions to form zinc phosphate (Zn_3_(PO_4_)_2_) precipitation, which is detrimental to the incorporation of phosphorus into ZnO nanorods [16]. However, the zinc nitrate being the strong acid and strong alkaline salt, it has a potential to minimize the possibility of zinc phosphate precipitation and hence can increase the probability of successful incorporation of phosphorus into ZnO nanorods [16]. The phosphorus doping in ZnO nanorods is known to induce p-type conductivity from their inherent n-type conductivity [7, 27, 28], which will further validate the doping of phosphorus atoms.

Using the Hall-effect measurements, we investigate the effect of phosphorus doping on electrical properties such as the type of conductivity, doping concentration, and mobility of charge carriers. In general, Hall-effect measurement of nanorods and/or nanowires is quite challenging due to its one-dimensional geometry. Thus, it is clear that the one-by-one Hall measurement of single nanorods is probably the most accurate one. However, the method is mostly valid to nanorods or nanowires of brittle and low density, which requires challenging processing procedures [45]. In this case, Hall-effect measurement is enabled by Al-doped ZnO seed layer beneath ZnO nanorods as a conducting medium. Due to the electrical imperfection of Al-doped ZnO seed layer as a medium for current flow, the measurement can possibly underestimate the actual electrical properties of the ZnO nanorods. However, the result yet can show how the NH_4_H_2_(PO_4_)_2_ M ratio changes the electrical properties of ZnO nanorods. The dependence of carrier concentration, Hall coefficient, and mobility on NH_4_H_2_(PO_4_)_2_ M ratio is illustrated in Fig. 3c, d, and e, respectively. The carrier concentrations for 0%, 0.05%, 0.1%, 0.2%, 0.5%, and 1% M ratios are − 6.1 × 10^15^, − 4.0 × 10^15^, − 3.4 × 10^15^, 1.6 × 10^15^, 7.8 × 10^15^, and 1.67 × 10^9^ cm^−2^, respectively. The negative sign in the doping concentration of the samples below 0.2% of the NH_4_H_2_(PO_4_)_2_ M ratio indicates their n-type conductivity, and the positive sign in the remaining samples reveals their p-type conductivity. Indeed, ZnO nanorods exhibit intrinsic n-type conductivity due to the presence of oxygen vacancy-related defects and/or Zn interstitials, yet the details are controversial [7, 27, 28]. However, with increasing NH_4_H_2_(PO_4_)_2_ M ratio, the ZnO nanorods are gradually being transformed into the p-type ones by compensating their intrinsic negative conductivity. The p-type conductivity by the incorporation of phosphorus is also observed in ZnO thin films [29–31]. On the other hand, the nanorods corresponding to 1% of NH_4_H_2_(PO_4_)_2_ M ratio showed quite different behavior as compared to the previous reports. As shown in Fig. 3c, the sample corresponding to 0.5% of NH_4_H_2_(PO_4_)_2_ M ratio showed the highest carrier concentration around 7.8 × 10^15^ cm^−2^ and falls down to 1.67 × 10^9^ cm^−2^ all of a sudden as soon as the NH_4_H_2_(PO_4_)_2_ M ratio was increased to 1%. We assume that this change is due to the amphoteric behavior of phosphorus in ZnO [27]. The phosphorus acts as either an acceptor or a donor depending on whether the phosphorus substitutes oxygen sites (P_O_) or Zn sites (P_Zn_), respectively. It is reported in [27] that the solubility of p-type dopants in ZnO is low. In these regimes, incorporation of excess phosphorus beyond the solubility limit, they substitute the Zn sites and compensate itself with P_O_ and hence can lose the p-type conductivity. The solubility limit of phosphorus is around 10^20^ cm^−3^ when Zn_3_P_2_ has been used for the purpose of phosphorus doping in the ZnO matrix [27]. However, we cannot clearly say how much is the solubility limit of phosphorus when it comes to growing p-type ZnO with NH_4_H_2_(PO_4_)_2_ via hydrothermal process, but we believe the solubility limit should be somewhere around 7.8 × 10^15^ cm^−2^. It is noteworthy that the carrier concentration can be increased by post-thermal annealing process as mentioned in [16]. However, the annealing process changes not only carrier concentrations but also their diameter, length, and density of the nanorods unexpectedly [16]. Thus, the annealing of the nanorods was not considered in the present work. The Hall coefficients (*R*_H_) for a semiconductor can be given by *R*_H_ *=* 1*/n*_c_*e* [32], where *n*_c_ represents the concentration of charge carriers, whose sign is negative and positive for n-type and p-type semiconductors, respectively, where the charge carriers are electrons and holes, respectively. The variation of *R*_H_ (shown in Fig. 3d) further confirms the transformation of conductivity from n-type to p-type in the ZnO nanorods. It is known that the Hall coefficient and mobility are related by the equation *μ = σR*_H_ [32], where *σ* represents the electrical conductivity. It may be noticed that the mobility is directly proportional to the Hall coefficients, and therefore, the variation of mobility as a function of the doping concentration also follows the nature of *R*_H_ curve (as shown in Fig. 3e).

**Fig. 4:**
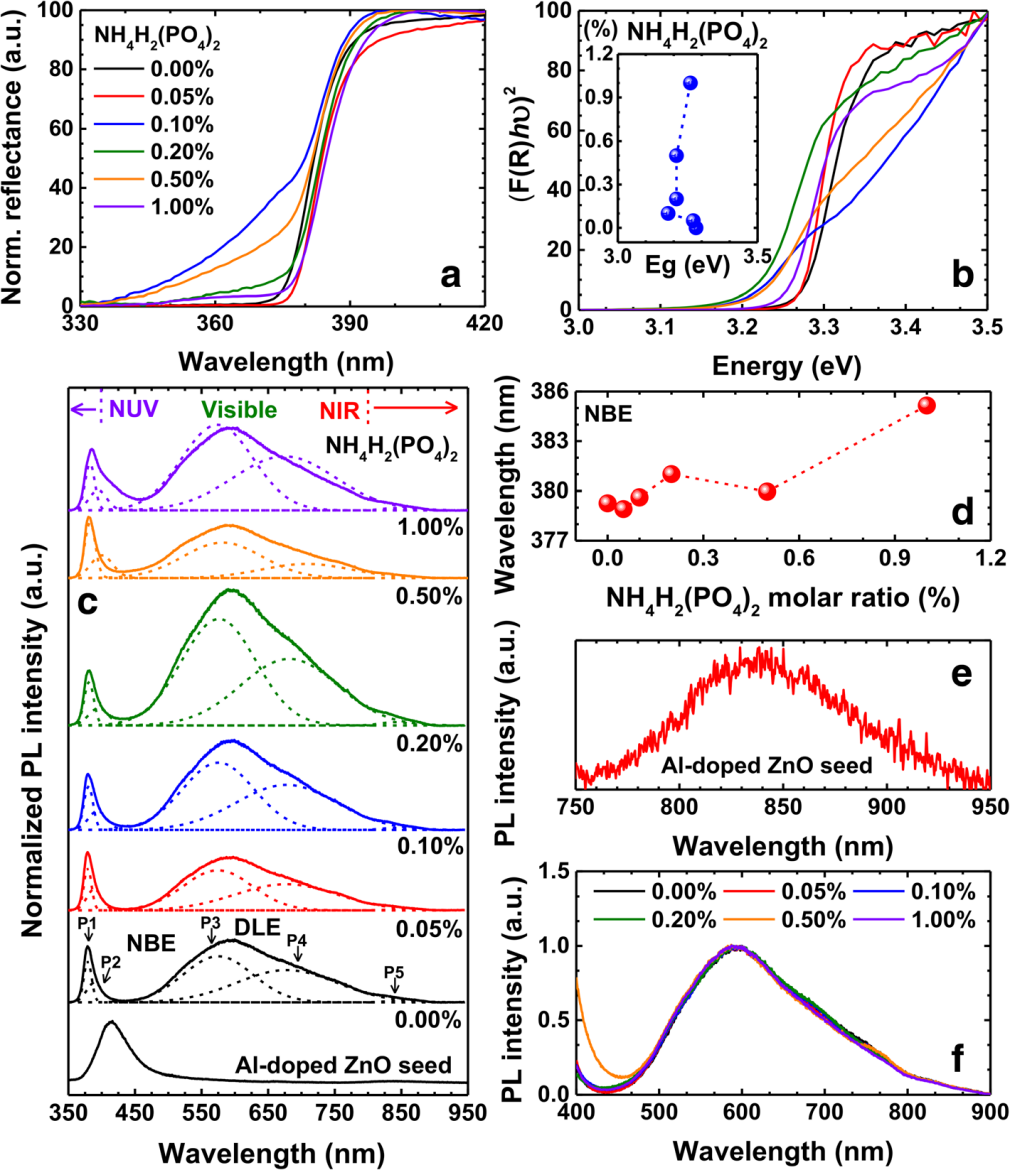
Normalized reflectance (**a**) and corresponding Tauc plots (**b**) for all the samples (inset: NH_4_H_2_(PO_4_)_2_ M ratio-dependent variation of optical bandgaps of ZnO nanorods.). **c** Normalized PL spectra of Al-doped ZnO seed layer, undoped ZnO nanorods, and phosphorus-doped ZnO nanorods. **d** The PL peak positions of NBE emission as a function of NH_4_H_2_(PO_4_)_2_ M ratio. **e** The magnified NIR emission from the Al-doped ZnO seed layer. **f** The DLE emission peaks of undoped and doped ZnO nanorods samples

Figure 4a shows the normalized reflectance of the undoped and phosphorus-doped samples, which were measured in the diffuse reflectance geometry. It is known that the sharp fall around 380 nm in the reflectance spectra indicates the optical bandgap of the ZnO samples. The tailing effect after the doping can be noticed in the sharp fall, which denotes a change in the optical bandgap due to the doping of phosphorus into ZnO nanorods. In order to determine the optical bandgap of these samples, we have used the Kubelka-Munk (KM) function, which was obtained from the reflectance spectra. The relation between the KM function (*F*(*R*)) and reflectance is given by *F*(*R*) *=* (1*−R)*^2^/2*R* [33], where *R* represents the reflectance of the samples, whose corresponding KM function has been plotted using the Tauc relation (shown in Fig. 4b). The optical bandgaps of all the samples were estimated from these Tauc plots, which are shown in the inset of Fig. 4b. The bandgap of undoped ZnO sample was found to be 3.28 eV, which reduces to 3.18 eV till the NH_4_H_2_(PO_4_)_2_ M ratio of 0.1%, and then, the bandgap increases above this concentration, which reaches 3.26 eV for the case of 1% NH_4_H_2_(PO_4_)_2_ M ratio. We note here that the bandgap of all the samples lies within the range of 3.18 and 3.28 eV. Though the bandgap of the ZnO nanorods is obtained from the Tauc plot, however, it deviates as per changes in NH_4_H_2_(PO_4_)_2_ M ratio. Indeed, obtaining the bandgap from the Tauc plot is probably not a proper way for the samples investigated in this article; this is because the Tauc plot ignores excitonic effect. In order to address this issue, we have performed the PL measurements on the all samples [49].

Figure 4c shows the normalized PL spectra of undoped and phosphorus-doped ZnO nanorods as well as Al-doped ZnO seed layer. It may be observed that all the spectra consist of two prominent peaks, one in the UV region and the other one being located in the region that covers visible and NIR regimes. It is known that the first peak in the UV region is related to the near-band-edge emission (NBE) and the other peak/hump is related to the deep level emission (DLE) in the ZnO nanorods. We note here that the origin of the deep level emission in ZnO is controversial and is expected to arise from various kinds of defects and/or vacancies [34–36]. Therefore, the peaks were deconvoluted carefully by considering the asymmetry in these spectra as shown in Fig. 4c, which provides an insight into the origin of the observed emissions. It is to be noted here that the deconvoluted peaks correspond to the UV, violet, yellow, red, and NIR emissions. The UV emission (P1) at ~ 379 nm in the undoped ZnO sample corresponds to their bandgap (as discussed above). This emission represents the characteristic feature of ZnO, which arises due to free excitonic transitions [14]. It is noteworthy that the bandgap obtained by PL is 10 meV smaller than the one of the Tauc plot (Fig. 4b). For example, the bandgap of undoped ZnO nanorods from PL is 3.27 eV corresponding to the 379-nm emission, and the one from the Tauc plot is 3.28 eV. This is presumably due to the Stokes shift [48]. As the doping concentration increases from 0 to 1%, this emission undergoes a bathochromic shift from 379 to 384 nm (as shown in Fig. 4d). According to the previous reports, the phosphorus doping induces an emission at ~ 384 nm, which is due to donor-acceptor pair (DAP) transitions [14, 25]. Therefore, the red shift in the present case can be attributed to the phosphorus-induced DAP emission in the ZnO nanorods [8, 14]. It is known that the diameter of the nanorods also affects the emission wavelength regarding the surface-to-volume ratio-dependent number of the quasi-Fermi level and the shift gets severe once the diameter is larger than 150 nm [44]. However, the largest diameter of the nanorods investigated is around 150 nm, and the rest of them are below 150 nm in this article; thus, we rule out the effect of the diameter change. The violet emission (P2) observed at ~ 389 nm (in undoped ZnO nanorods sample) is due to Zn interstitials, whose emission also undergoes a red shift, from 389 to 408 nm, after the doping [37]. The observed yellow emission (P3), within the wavelength range of 574–587 nm, is due to the presence of interstitial oxygen atoms [38, 39]. The presence of excessive oxygen or zinc vacancies is responsible for the observed red emission (P4) [40, 41], which covers the wavelength range of 678–729 nm (as shown in Fig. 4c). It may be observed that the full width at half maximum (FWHM) of the yellow and red emissions is much higher compared to the other emissions. We note here that the deconvolution made was solely based on the observed asymmetry of the peaks and it may happen that these two peaks might consist of one or more peaks. Therefore, one cannot exclude the possibility of the existence of green and orange emissions within the aforementioned yellow and red emissions, respectively. On the other hand, the emission (P5) in the NIR region was found to show no significant change in both the position and FWHM of the peaks as a function of doping, whose variation lies within the error bars (not shown here). We note here that the only common constant factor in all these samples is the seed layer, which is Al-doped ZnO film in the present case. Moreover, the PL spectrum of the seed layer alone (Fig. 4c, e) does confirm the NIR emission as expected, which may be noticed in Fig. 4e. Furthermore, the PL spectrum from the seed layer shows another emission at 425 nm (Fig. 4c), which is the characteristic NBE emission of the Al-doped ZnO seed layer [42]. However, the reason for the NIR emission from the Al-doped ZnO thin films remains to be understood. It is to be noted here that the peak positions of deep level emissions do not undergo any significant change as a function of doping concentration while varying NBE emission changes, as shown in Fig. 4f. The persistent peak wavelength regardless of NH_4_H_2_(PO_4_)_2_ M ratio can be advantageous in designing visible light-emitting devices which utilize the DLE emission. Let's consider a simple visible light-emitting device structure which is composed of phosphorus-doped p-type ZnO nanorods and n-type substrate, the p-n junction. In that case, the phosphorus-doped p-type ZnO nanorods should be not only a light emitting medium but also an electrical carrier injection medium. To be an efficient electrical carrier injection medium, it is needless to say that the phosphorus-doped ZnO nanorods should be a highly doped ones. In such a circumstance, let’s assume another condition that the DLE emission wavelength of phosphorus-doped ZnO nanorods depends on phosphorus concentration and/or carrier concentration. Then, the emission wavelength is compelled to pin to a certain emission wavelength of highly phosphorus-doped ZnO nanorods. This is because, we have no other choice but to keep the carrier concentration as high as possible to have an efficient carrier injection medium. However, unfortunately, the emission wavelength of highly phosphorus-doped ZnO nanorods can possibly not matches with the target emission wavelength we expect from the light-emitting devices; failing in light-emitting device design. Also, in real world, the visible DLE emission wavelength of phosphorus-doped ZnO nanorods does not change as per carrier concentration as shown in Fig. 4f. Then how can we tune the emission wavelength? Indeed, there are more parameters to consider in designing the light-emitting devices yet, in other words, the parameters  to tailor DLE emission wavelength. Simimol et al. [43] and other literature indicate that the ZnO nanorods upon annealing changes the emission wavelength and hence can serve the purpose of tuning the emission spectrum. In that case, the persistent DLE emission wavelength of phosphorus-doped ZnO nanorods as per carrier concentration enables designing the light-emitting device rather straightforward; we have only one parameter (annealing) to consider in tailoring emission wavelength, and another one (phosphorus concentration or NH_4_H_2_(PO_4_)_2_ M ratio) in electrical carrier injection, separately.  Such an approach will make phosphorus-doped ZnO nanorods as a platform to fabricate light-emitting devices *a la carte* in visible wavelength range with the cheapest route along with the hydrothermal process. In addition, we further note here that the observed emissions in the most important regimes of the electromagnetic spectra including UV and visible emission range would be interesting for a broad range of applications from biological to optoelectronic devices. However, it is noteworthy that the persistent p-type doping in ZnO nanorods as well as thin films is yet challenging for practical device applications. In other words, though a 16-month-long p-type conductivity of phosphorus-doped ZnO is quite persistent [14], yet not comparable to the other inorganic crystalline semiconductors such as GaN: Gallium nitride, GaAs: Gallium arsenide, and InP: Indium phosphide. The unstable p-type conductivity is originated by intrinsic native defects [46, 47], and further study should be addressed on the precise control of the defects.

## Conclusions

In summary, the p-type conductivity in ZnO nanorods has effectively been accomplished by the doping of phosphorus impurities into them. The successful doping of phosphorus into ZnO nanorods enhances the length and diameter of the nanorods. An unusual variation of carrier concentration, mobility, and Hall coefficient as functions of NH_4_H_2_(PO_4_)_2_ M ratio i.e. phosphorus concentration was noticed, which was explained on the basis of the amphoteric nature of phosphorus. These hydrothermally synthesized ZnO nanorods grown on Al-doped ZnO seed layer were found to show PL emission in the three different regimes including UV, visible, and NIR regimes. The observed emissions in UV, violet, yellow, red, and NIR regimes were attributed to NBE emission, zinc interstitials, oxygen interstitials, excess oxygen (or zinc vacancies), and characteristic feature of Al-doped ZnO seed layer, respectively. Interestingly, the doping of phosphorus into these nanorods led to a change in the UV emissions and does not affect the visible and NIR emissions. Such unusual effects in ZnO by phosphorus incorporation can be suitable for various optoelectronic and biological applications.

## Abbreviations

DLE: Deep level emission
HMTA or C_6_H_12_N_4_: Hexamethylenetetramine
KM method: Kubelka-Munk method
NBE: Near-band-edge emission
Nd-YAG: Neodymium-doped yttrium aluminum garnet
NH_4_H_2_(PO_4_)_2_: Ammonium dihydrogen phosphate
NIR: Near-infrared
PL: Photoluminescence
P_O_: Oxygen sites in ZnO
P_Zn_: Zinc sites in ZnO
RF: Radio frequency
SEM: Scanning electron microscope
UV: Ultraviolet
XRD: X-ray diffraction
Zn(NO_3_)_2_: Zinc nitrate hexahydrate
ZnO: Zinc oxide
